# Advances in Enhancing the Photothermal Performance of Nanofluid-Based Direct Absorption Solar Collectors

**DOI:** 10.3390/nano15181428

**Published:** 2025-09-17

**Authors:** Zenghui Zhang, Xuan Liang, Dan Zheng, Jin Wang, Chungen Yin

**Affiliations:** 1 School of Energy and Environmental Engineering, Hebei University of Technology, Tianjin 300401, China; zzh_jjwg@163.com (Z.Z.); liangxuan3452024@163.com (X.L.); 2 School of Engineering, University of British Columbia, Kelowna, BC V1V 1V7, Canada; 3 Center for High Reliability Power Semiconductor, Yangtze Delta Region Institute of Tsinghua University, Jiaxing 314001, China; 4 Department of Energy Technology, Aalborg University, 9220 Aalborg East, Denmark; chy@energy.aau.dk

**Keywords:** nanofluid, heat transfer enhancement, optical absorption, solar collector, photothermal conversion

## Abstract

The integration of nanofluids into solar collectors has gained increasing attention due to their potential to enhance heat transfer and support the transition toward low-carbon energy systems. However, a systematic understanding of their photothermal performance under the direct absorption mode remains lacking. This review addresses this gap by critically analyzing the role of nanofluids in solar energy harvesting, with a particular focus on the direct absorption mechanisms. Nanofluids enhance solar radiation absorption through improved light absorption by nanoparticles, surface plasmon resonance in metals, and enhanced heat conduction and scattering effects. The novelty of this work lies in its comparative evaluation of advanced nanofluids, including magnetic nanofluids, plasma nanofluids, and nanophase change slurries, highlighting their unique capabilities in flow manipulation, thermal storage, and optical energy capture. Future research directions are identified, such as the life cycle assessment (LCA) of nanofluids in solar systems, applications of hybrid nanofluids, development of predictive models for nanofluid properties, optimization of nanofluid performance, and integration of Direct Absorption Solar Collectors (DASCs). In addition, challenges related to the stability, production cost, and toxicity of nanofluids are critically analyzed and discussed for practical applications. This paper offers guidance for the design and application of high-performance nanofluids in next-generation solar energy systems.

## 1. Introduction

The global energy landscape in 2022 continued to be shaped by multiple factors, including the ongoing recovery from the COVID-19 pandemic, the persistent impact of supply chain issues, and the geopolitical instability caused by the conflict in Ukraine. Fossil fuels remain the dominant source of global energy, accounting for 82% of primary energy. However, carbon dioxide emissions reached a record high of 39.3 billion tonnes, with 34.4 billion tonnes coming from energy use alone. At the 28th Conference of the Parties to the United Nations Framework Convention on Climate Change, experts emphasized the importance of renewable energy, hydrogen, carbon capture and storage, and other low-carbon technologies in achieving a net-zero emissions future.

Most countries are currently undergoing a transition from traditional energy sources to newer forms of energy. This transition requires a significant amount of time and effort to alter the global energy structure. A critical strategy during this transformation is the development of an efficient, integrated innovative energy system, which can drive progress toward the “dual carbon” goal of carbon peak and carbon neutrality. Achieving carbon neutrality requires the replacement of fossil fuels with electricity and clean energy sources, such as solar, wind, and hydro power, for electricity generation. Furthermore, integrating novel thermal media into energy systems can enhance energy conversion, storage, and distribution, contributing to overall system performance improvements.

**Figure 1:**
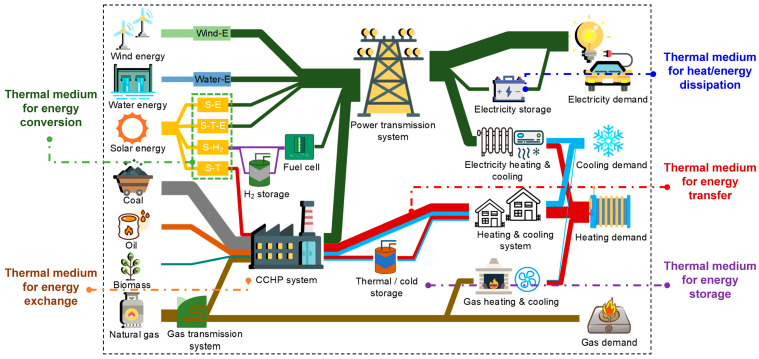
A presupposed multiple energy system for “carbon neutrality”.

A comprehensive innovative energy system, as illustrated in Figure 1, integrates multiple energy sources and conversion pathways, including wind, solar, and hydroelectric power, which are converted into electricity, hydrogen, and thermal energy. These energy forms are then distributed through grids, networks, and pipelines to meet various demands. The application of thermal media at different stages of the energy system improves efficiency and coordination. During energy conversion, thermal media such as steam and thermal fluids enhance the utilization of solar heat. In energy exchange, thermal media facilitate the multiform energy output seen in combined cooling, heating, and power systems. Additionally, thermal media ensure stable energy transfer in district heating and cooling networks and enable efficient energy storage using phase change materials. By reducing energy losses and enhancing system flexibility, this multi-level integration of renewable energy and thermal media is a practical approach to low-carbon, efficient production.

The transition to renewable energy has gained momentum, with solar and wind energy experiencing a record increase in capacity in 2022. Solar energy, in particular, accounted for 72% of the 266 GW added globally. Photothermal conversion, a key technology in solar energy utilization, accounts for approximately 70% of solar energy applications. Solar collectors, which convert solar radiation into thermal energy, play a central role in photothermal applications. However, the slow development of photothermal systems in many regions is primarily due to low conversion efficiency and limited solar radiation absorption.

In recent years, the performance of solar energy systems has been significantly enhanced by advances in nanofluids, an emerging class of engineered colloids. First introduced by Choi et al. in 1995 [1], nanofluids are colloidal suspensions consisting of nanoparticles (typically < 100 nm) dispersed in conventional base fluids such as water, ethylene glycol, or oils. Due to their colloidal nature, nanofluids exhibit enhanced thermal transport characteristics resulting from the synergistic effects of Brownian motion, thermophoresis, and nanoparticle-fluid interfacial interactions. These features significantly boost thermal conductivity, convective heat transfer, and even radiative properties [2], making nanofluids highly promising for solar thermal applications. In addition to solar energy applications, nanofluids have shown promise in various fields, including biomedicine [3] and microfluidics [4]. These diverse applications highlight the broad potential of nanofluids beyond energy harvesting.

As a subclass of colloids, nanofluids benefit from colloidal stability principles to maintain uniform dispersion, which is critical for consistent thermal performance. Advances in surfactant chemistry and nanoparticle surface functionalization have improved the long-term stability of these colloids under varying thermal and flow conditions. Such stability is crucial for their integration into solar collectors, including flat-plate collectors [5], evacuated-tube collectors [6], and parabolic-trough collectors [7]. These colloidal systems enhance energy absorption while simultaneously supporting compact, high-efficiency designs with minimized heat losses.

**Figure 2:**
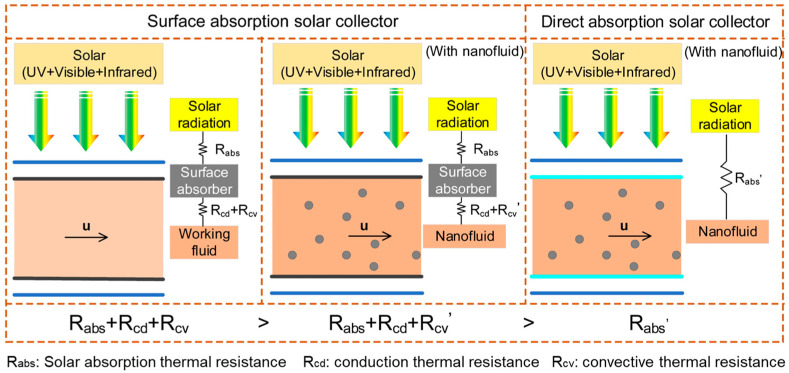
Comparison of thermal resistance for solar collectors with different radiation absorptions.

Figure 2 compares the heat transfer mechanisms in surface absorption and direct absorption solar collectors. In conventional surface absorption solar collectors, solar radiation is first absorbed by a solid surface and then transferred to the working fluid through conduction and convection, resulting in a total thermal resistance of Rabs + Rcd + Rcv. The utilization of nanofluids in such systems enhances thermal conductivity and promotes micro-convection due to nanoparticle movement, thereby improving heat transfer performance. However, energy loss still occurs during interfacial heat transfer. In contrast, direct absorption solar collectors utilize nanofluids as both the heat transfer medium and the solar absorption medium. The incident solar radiation is volumetrically absorbed by the suspended nanoparticles, eliminating interfacial conduction and convection resistances. This leads to a reduced total thermal resistance and significantly enhances photothermal conversion efficiency. As illustrated in Figure 2, the transition from surface absorption to direct absorption with nanofluids results in a more efficient solar energy harvesting process, as it minimizes thermal losses.

Despite significant advancements in nanofluid-based solar collectors, there is a lack of comprehensive studies addressing the specific challenges of applying nanofluids in various types of solar collectors. Current research has mainly focused on surface absorption systems, with less attention given to the integration of nanofluids in direct absorption systems. Moreover, while the thermophysical properties of nanofluids are well-documented, there is limited understanding of how these properties interact with the operational conditions and structural designs of solar collectors. This knowledge gap presents an opportunity for further research to optimize nanofluid formulations and explore their applications in various solar collector technologies, ultimately enhancing the efficiency of solar energy systems.

This review enhances the understanding of the mechanism by which nanofluids improve heat transfer and optical absorption, and aims to further improve photothermal conversion performance through the adjustment of nanomaterials based on a mechanistic analysis. To mitigate the impact of the collector structure on photothermal performance, this paper presents a comparative analysis of the light absorption performance of nanofluids in a volume absorption solar collector. Moreover, nanofluid-based direct absorption shows promising opportunities for reliable operation in real-world applications, such as solar steam generation, photocatalytic hydrogen production, and thermal energy storage, highlighting its potential for translation to industrial-scale systems.

## 2. Application of Nanofluids in Solar Radiation Direct Absorption

**Figure 3:**
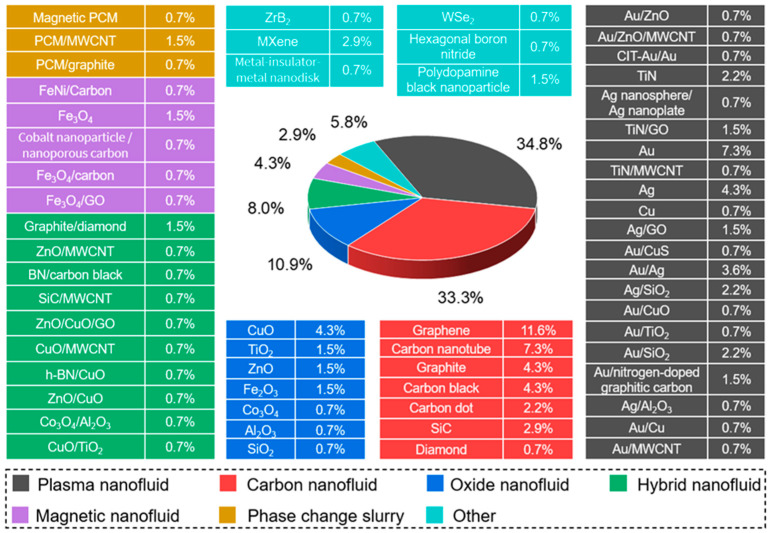
Proportion of various nanomaterials in solar radiation direct absorption (Note: publications in the Web of Science database from 2018 to 2022, keywords of “Direct absorption solar collector” and “nanofluid”, total 132 papers).

Direct absorption solar collectors (DASCs) employing nanofluids as the working medium offer superior photothermal conversion efficiency compared to traditional surface absorption collectors [8,9]. In DASCs, the thermophysical properties and optical absorption characteristics of the nanofluid critically influence overall thermal performance. Functional nanofluids, which incorporate advanced nanomaterials, have demonstrated substantial potential for improving energy conversion efficiency. Figure 3 presents the distribution of nanomaterial species used in DASC research, based on publication data. Carbon-based nanomaterials account for 33.3% of reported applications, with graphene (11.6%) and carbon nanotubes (7.3%) being the most frequently studied due to their excellent thermal conductivity and ability to absorb broadband light. Metal-based nanomaterials also comprise a large share (34.8%), reflecting their strong plasmonic absorption and compatibility with solar spectra. Hybrid, oxide, magnetic, phase change, and plasma nanofluids have been explored to enhance thermal performance through synergistic or multifunctional effects. These trends highlight the ongoing shift toward engineered nanofluids with tailored properties for advanced solar thermal applications.

### 2.1. Oxide Nanomaterial-Based Nanofluid

**Figure 4:**
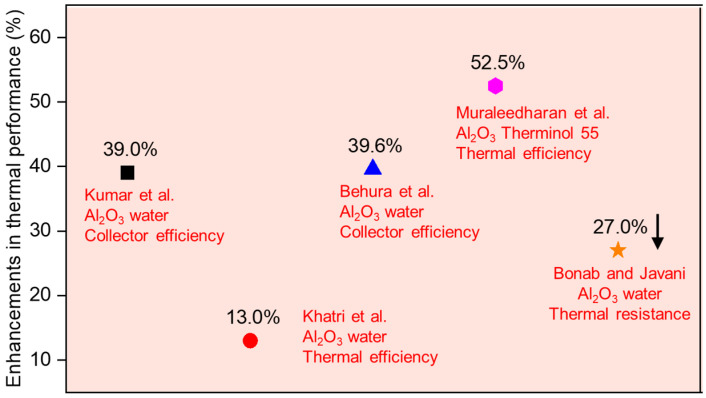
Comparison of enhancements in photothermal performance of the DASCs with Al_2_O_3_ nanofluids.

Compared with surface absorption solar collectors, metal oxide-based nanofluids are rarely investigated in direct absorption solar collectors due to the low competitiveness of metal oxide nanoparticles in light capture. Some studies about oxide nanomaterials, such as CuO and Al_2_O_3,_ have been conducted in direct absorption solar collectors. According to the relevant results, the Al_2_O_3_ nanofluid significantly improves solar collector efficiency in the range of 13% to 52.2% compared to base fluids, as shown in Figure 4. On improvements in instantaneous efficiency for direct absorption solar collectors, 52.2% and 39.6% increases are reported for 0.1 vol.% Al_2_O_3_ [10] and 0.005 wt.% Al_2_O_3_ nanofluids [11]. For CuO nanofluids, the extinction coefficient increases by 176% with the increase in nanoparticle concentration from 0.01 vol% to 0.1 vol% [12]. The combined application of CuO nanofluids and spectrally selective coatings in the absorber tube results in a 31.2% increase in thermal efficiency for a direct absorption solar collector [13]. The maximum and minimum observed radiation at the specified position were 1000 W/m^2^ and 550 W/m^2^, respectively, with each experiment lasting 6 h (from 10:00 to 16:00). Temperatures and solar direct radiation were recorded every 5 min, with a 30 min average value provided. The high scattering properties of Al_2_O_3_ nanoparticles and the high absorption properties of CuO nanoparticles are utilized to prepare binary nanofluids, resulting in a 50% increase in thermal efficiency for a direct absorption parabolic trough collector [14]. A comparison is conducted between water-based Al_2_O_3_ and CuO nanofluids, and the CuO nanofluid shows nearly a threefold increase in absorbance compared to the Al_2_O_3_ nanofluid in the visible range at a 0.02 wt.% concentration [15]. This study was conducted under an average maximum solar intensity of 999.16 W/m^2^, which contributed to the enhanced performance of the nanofluid. The CuO and Al_2_O_3_ nanofluids with volumetric fractions below 5% show better efficiency in the solar volumetric absorption system compared to high-volume fractions [16]. The tests were conducted with an inlet temperature of 380 K, an ambient temperature of 300 K, and solar irradiance of 1000 W/m^2^, which allowed for a significant improvement in the absorbance of CuO nanofluids. High nanoparticle concentrations increase heat loss due to non-uniform temperatures at the receiver tube [17]. The optical penetration distance is short for the nanofluid with a high nanoparticle concentration, and the incident radiation is absorbed in a thin fluid layer adjacent to the tube wall. The near-wall overheating intensifies convective and radiative heat loss to the surroundings.

Nanoparticles of Fe_3_O_4_ are often used in industrial applications due to their thermomagnetic effect. Studies have concluded that the magnetic field in magnetic nanoparticles improves the convective heat transfer performance of a nanofluid by approximately 13–75% [18]. As a thermoelectric power output enhancer, the maghemite nanoparticle is an excellent absorber of solar radiation [19]. To achieve optimal photothermal performance, thermomagnetic convection compensates for the weakening of natural convective heat transfer when the solar angle is low [20]. The photothermal conversion efficiency of DASCs increases by employing magnetic nanofluids under an external magnetic field [21]. This enhancement is attributed to the effect of magnetic nanoparticles as nano-rotors. The contribution of morphology to improving light absorption capability is assessed for Fe_2_O_3_ nanoparticles in direct absorption solar collectors. 96.2% absorption of incident solar energy for the blade-shaped Fe_2_O_3_ nanofluid is higher than 93.5% for the spherical-shaped Fe_2_O_3_ nanofluid and 87.2% for the octahedral-shaped Fe_2_O_3_ nanofluid. The highest solar-thermal conversion efficiency of 82% is obtained for 0.02% blade-nanofluid [22]. The influence of morphology on the efficiency of nanofluids is multifaceted. Generally, nanoparticles with elongated shapes or larger surface areas (such as blade-shaped and rod-shaped particles) provide higher light absorption efficiency and thermal conductivity, thus enhancing photothermal conversion efficiency. However, issues related to aggregation and stability of these particles need to be effectively managed. Therefore, the shape of nanoparticles, along with their dispersion, aggregation, and light and heat conduction properties in the fluid, are closely interconnected and determine the overall performance of nanofluids.

### 2.2. Carbon/Nitrogen Nanomaterial-Based Nanofluid

Compared with other nanomaterials, high collector efficiency is generally achieved by the nanofluid with carbon nanomaterials, such as carbon black, carbon soot, multi-walled carbon nanotube (MWCNT), and graphene [23]. This result is caused by the high optical absorption performance, high thermal conductivity, and long stable time [24]. A photothermal efficiency of up to 122.7% is achieved using the carbon-based nanofluid with a low concentration of at least 0.01 vol.% in direct absorption solar collectors [25].

The ethylene glycol (EG)-based nanofluid with MWCNTs exhibits excellent optical absorption performance, particularly in the 300–700 nm spectral range. With an MWCNT concentration above 0.005 wt%, the penetration distance exceeds 1 cm. The solar weight absorption rate converges to 1, indicating that solar energy is almost completely absorbed [26]. Efficiencies of up to 56.7% and 66% are observed for collectors using water and a water-based nanofluid with 0.2 vol.% single-walled carbon nanotubes (SWCNTs) [27].

Graphite nanopowders increase the absorption coefficient of Therminol VP-1 by more than eight times when the wavelength range increases from 350 to 1600 nm [28]. Thermophysical properties and dispersion stability are significantly affected by the surface modification of graphene nanomaterials. The differences among various treatment methods are first reflected in the thermal conductivity of nanomaterials. The addition of reduced graphene oxide (RGO) powders treated with tetra ethyl ammonium hydroxide results in a 9.23% reduction in thermal conductivity compared to pure water at 40 °C. The thermal conductivity of the graphene oxide-based aqueous nanofluids without treatments is higher than that of pure water [29]. The photothermal conversion efficiencies increase by 172% and 189% for single-layer graphene (SLG) and graphene oxide-based nanofluids compared with pure water, corresponding to photothermal conversion efficiencies of 46.26% and 49.13% [30]. Compared with the deactivated nanofluids, the RGO/water-ethylene glycol (EG) nanofluid shows excellent dispersion stability at high temperatures [31]. The two-dimensional sheet morphology of the RGO provides a large aspect ratio, which reduces re-agglomeration. Additionally, the residual oxygen-containing functional groups on RGO sheets contribute to electrostatic/steric repulsion, resulting in high wettability. In addition, strong hydrogen bonding and dipole interactions between RGO and the water–EG binary solvent further stabilize the suspension, and the high thermal conductivity of RGO helps to alleviate localized overheating that usually promotes aggregation. These combined effects enable the RGO/water–EG nanofluid to maintain uniform dispersion even under thermal stress, in contrast to the rapid destabilization observed in deactivated nanofluids. After 1000 s irradiation, the photothermal conversion efficiencies of Ti_3_C_2_/[BMIM]BF_4_ nanofluid are 20% and 29% higher than those of GO/[BMIM]BF_4_ and MWCN/[BMIM]BF_4_ nanofluids in a DASC, owing to the outstanding broad-spectrum absorption properties of Ti_3_C_2_ (250–2100 nm) [32]. An increase of 55.47% in intercept efficiency is achieved for the water-based nanofluid with a two-dimensional layered nanostructure MXene consisting of an Al layer and a Ti_3_C_2_ layer (at a mere 0.01 wt.%) [33]. The performance of the direct absorption solar collector is determined by the optical properties of the working medium.

Compared with carbon nanostructures, common carbon nanomaterials significantly reduce the preparation cost. Based on the measurement of solar-weighted absorption fraction, the nanofluid with cost-effective carbon soot achieves 70% absorption of incident energy. This result leads to a 37.6% increase in thermal efficiency compared to the base fluid [34]. With high colloidal stability for six months and a low corrosion rate of 0.094 mm/year, the carbon dot/water nanofluid exhibits a maximum increase in thermal efficiency of 57.62% compared to water [35]. Carbon quantum dot (CQD) nanofluids achieve a triple solar thermal conversion efficiency of polyethylene glycol 200 as a base fluid. The solar intensity and working temperature have a minimal effect on solar thermal conversion efficiency due to the ultra-stability of the CQD nanofluid [36]. An activated carbon-based nanofluid with a 0.6% volume fraction achieves a receiver efficiency of 94.51% after 600 s of radiation [37]. The EG-based nanofluid with boron nitride/carbon black exhibits a 34.55% increase in photothermal conversion efficiency compared to EG under 1200 s of exposure to 437 W/m^2^ incident irradiance [38]. The silicon carbide semiconductors show great potential in direct absorption solar collectors. For example, DI-water with 0.004 vol.% SiC nanoparticles exhibits an 81.6% improvement in photothermal conversion efficiency [39]. The SiC porous foam, with a porosity of 90% and a pore diameter of 20 PPI, exhibits a four times higher extinction coefficient than the DI-water-based nanofluid with 0.1 vol.% foam [12].

**Figure 5:**
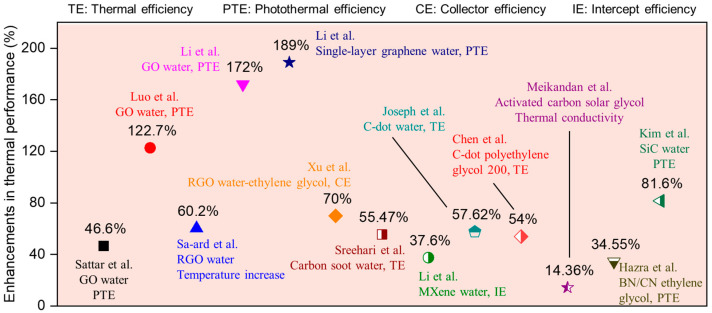
Comparison of enhancements in photothermal performance of the DASCs with carbon-based nanofluid.

The improvements in thermal performance are summarized in Figure 5. Compared with conventional nanofluids, carbon/nitrogen nanomaterial-based nanofluids provide a higher potential in photothermal conversion, as listed in Table 1. High thermal conductivity, a large specific surface area, and excellent optical absorption capacity significantly enhance the performance of carbon/nitrogen nanomaterial-based nanofluids. The optical absorption performance of the EG-based TiN nanofluid is tested at various temperature values. Results indicate a 50% increase in optical absorption performance from 0 to 60 °C. The selected test temperature does not correspond to the operating temperature of the solar collector. Hybrid nanofluids comprising various nanoparticles have been utilized to enhance the absorption efficiency of direct absorption solar collectors [40]. The boron nitride (h-BN)/CuO nanocomposite is prepared by hydrothermal synthesis. The nanofluid with an h-BN/CuO ratio of 0.75:1 exhibits a photothermal conversion efficiency of 44.83%, surpassing that of pure water [41]. The thermal performance of the base fluid is often improved by adding carbon materials and other nanoparticles simultaneously [42]. Compared with the base fluid, the boron nitride (BN)-carbon black nanofluid shows a 34.55% increase in photothermal conversion efficiency. More than 40% solar-thermal conversion efficiency is achieved for SiC-MWCNTs and hydroxylated graphene nanofluids. The Al_2_O_3_-MWCNTs nanofluid contributes to a 197.1% increase in thermal efficiency [43]. The magnetic properties of graphene oxide materials are enhanced through the doping modification of α-Fe_2_O_3_ nanoparticles, resulting in a 14.5% improvement in photothermal conversion efficiency under a rotating magnetic field [44]. Some studies have pointed out that the light-to-heat conversion properties of hybrid nanofluids show few advantages or disadvantages compared to those of single nanofluids [9]. The performance is related to particle concentration, dispersion status, fluid type, and other factors.

### 2.3. Plasmonic Nanofluid

The plasmonic nanofluid has been introduced to enhance light absorption in solar thermal utilization [47]. Low additives are loaded into plasmonic nanofluids, which are utilized to achieve high photothermal conversion efficiency in direct absorption solar collectors [48,49]. Nanoparticles with sufficiently low concentrations further avoid particle aggregation, which maintains the stable performance of solar collectors [50].

**Figure 6:**
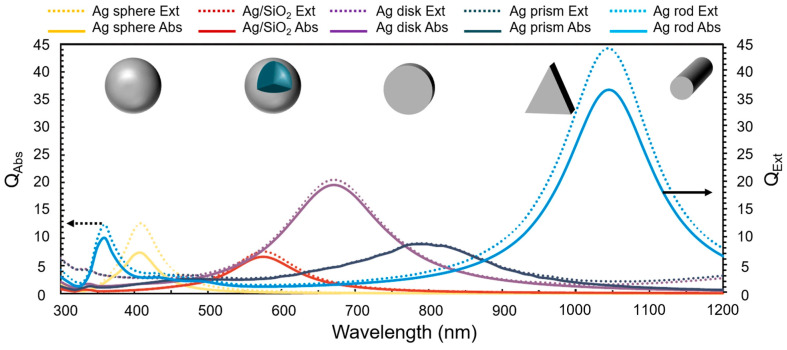
Spectral absorption range of Ag nanomaterials with different morphologies [53].

The absorption peak of precious metals, such as gold, silver, and copper, is usually located in the visible part [51,52]. Generally, the absorption band is narrow due to the localized surface plasmon of a single-type nanomaterial. The shortage of narrow absorption bands has been overcome by developing composite nanoparticles with multiple absorption peaks at different wavelengths. The spectral absorption and scattering efficiency of plasmonic nanofluids are controlled by tailoring the morphologies of plasmonic nanoparticles, as shown in Figure 6 [53]. Fusiform CuO/Ag plasmonic nanoparticles have remarkable broadband absorption with three peaks in the visible and near-infrared regions [54]. Ag nanoparticles with sharp edges induce multiple absorption peaks due to the localized surface plasmon resonance and the lightning rod effect [55]. In addition to the anisotropic particle morphologies, the enhancement of photothermal conversion efficiency also depends upon the size distributions of various nanoparticles. The photothermal characteristics of the nanofluid are mainly affected by the larger-sized silver nanoparticles in the range [56]. Table 2 shows a summary of studies for optical characterization of plasmonic nanoparticles.

To broaden the absorption band, nanoparticles with a core/shell structure increase the solar radiation absorber coefficient compared to solid nanoparticles. It is necessary to fabricate colloids of solid particles with 18% nanoshell and 53% multilayer structures for the metal materials [57]. These structures significantly impact the near-field radiative properties of both composite nanoparticles and plasmonic nanofluids [58]. For the core/shell nanocomposites, the dispersion stability and absorption coefficient are improved by investigating the effects of core size, shell thickness, and core/shell components [59,60]. Free surfactants are typically dispersed in base fluids to achieve a stable nanoparticle suspension; however, the nanofluid is prone to instability after prolonged exposure to direct sunlight. The plasmonic nanofluids, comprising synthesized Ag@SiO_2_ core/shell nanoparticles, exhibit 100% dispersion stability over 6 months without the use of any surfactants [61]. The chemical and colloidal stability of nanofluids containing citrate- (CIT-) and polyethylene glycol-coated (PEG-) gold nanoparticles is verified for 16 months of ambient storage [62]. The dispersion stability is retained for over three years for gold nanoparticles with polyvinylpyrrolidone as a soft protectant shell [63]. Subtle physicochemical transitions in polymeric coatings affect the optical properties and wettability of the nanofluids [64]. The nanoshells serve as anti-corrosion encapsulants to increase the intensity of the localized plasmon resonance and the absorption efficiency [48,65]. For an equal temperature rise, the SiO_2_/Au core/shell nanostructure-based nanofluid is only 1/5 the volume fraction of the Au nanofluid due to the morphology modification of nanocomposites [49].

**Table 2:** Summary of studies for optical characterization of plasmonic nanoparticles.

Authors (Year)	Nanoparticle/Base Fluid	Nanoparticle Size	Remarks
Sharaf et al. (2019) [62]	Au/DI-water	10 nm	Sixteen-month stability for citrate- (CIT-) and polyethylene glycol-coated (PEG-) gold nanofluids
Joseph et al. (2019) [66]	SiO_2_-Ag, CuO/water	300 nm	An 82.82% solar-weighted absorption fraction for hybrid nanofluid (SiO_2_/Ag: 206.3 mg L^−1^, CuO: 864.7 mg^−1^, and SDS: 1996.2 mg^−1^)
Sreekumar et al. (2020) [51]	Antimony tin oxide- silver nanoparticle/DI-water	Less than 10 nm	A 98.90% solar-weighted absorptivity for 0.2 wt% nanofluid; A 63.5% thermal efficiency for nanofluid-seeded parabolic through direct absorption solar collector at a flow rate of 0.022 kgs^−1^
Kumar et al. (2020) [47]	Au/DI-water	40–45 nm	A 33% increase in optical efficiency of gold plasmonic nanofluid compared with DI water at 0.030 kgs^−1^ flow rate
Wen et al. (2021) [67]	TiN/ethylene glycol	20 nm	A 99% solar absorption ratio for TiN nanofluid; An increase of 67.1% in solar-thermal conversion efficiency for TiN nanofluid compared with glycol ethanol
Mallah et al. (2022) [68]	Ag/water	25–35 nm	An 85% collector efficiency for 0.94 ppm volume fraction of blended plasmonic nanofluid at irradiation of 949 Wm^−2^

**Figure 7:**
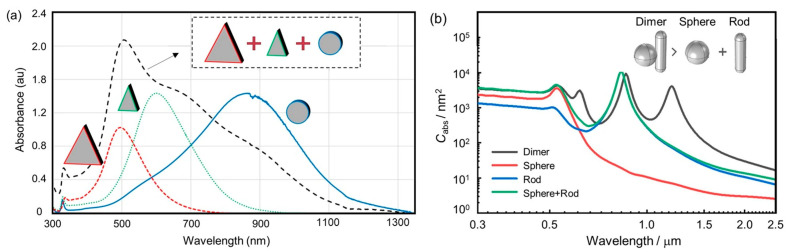
Spectral absorption characteristics of mixed and composite nanoparticles: (**a**) UV–Vis–IR spectra for silver nanofluids, all measurements undertaken with a 10 mm path length cuvette [73]; (**b**) absorption cross-section for different nanoparticles with sphere radius of 20 nm, rod radius of 10 nm, embedded depth of 1 nm, and rod length of 60 nm as default [52].

The coupled effect of different nanoparticles is investigated in a broad spectrum of solar radiation. The hybrid nanocomposite exhibits higher optical absorption than the single-particle nanomaterial at the same concentration, owing to the dual localized surface plasmon resonance effect [69]. To achieve a broad spectral absorptivity, silver nanoparticles with high absorption in the visible spectrum are combined with antimony-doped tin oxide, which exhibits an absorption peak in the near-infrared region [51]. High optical absorption features and low optical scattering properties are achieved when the gold nanoshells are doped with transition metal impurities, such as Pt, Fe, or Ag [70]. For the Cu@C core–shell nanoparticles, the solar absorption performance is enhanced due to the coupled effect of the local surface plasmon resonance of the Cu core and the strong intrinsic absorption of the carbon shell [60]. Anti-corrosion encapsulants of Al_2_O_3_ nano-layers improve the optical absorption by broadening the absorbance spectra of Ag nanoparticles [65]. It has been demonstrated that mixed nanofluids with distinct absorption peaks exhibit enhanced solar absorption performance [71]. The matching of the extinction spectrum and solar radiation spectrum is conducted by changing the proportion of components in the hybrid nanofluid. Based on the blended morphology, the full-spectrum absorption characteristics are achieved using the silica-coating-based nanofluid with Ag nanospheres and nanoprisms [48]. For the blended nanofluid, SiO_2_/Ag nanoshells with different core sizes and shell thicknesses are employed to further reduce the required volume fraction of Ag nanoparticles (10%) compared to the Ag nanofluid at the same temperature [59]. Gold nanoparticles (Au NPs) and ZnO particles with hedgehog-like hierarchical structures are used to prepare an oil-based nanofluid, which exhibits a 240% increase in photothermal conversion efficiency compared to the base fluid [72]. The full-spectrum absorption of the mixed nanofluid is based on the spectrum complementation of nanofluids, as shown in Figure 7a [73]. Compared with the binary nanofluid, the strong interaction of two nanomaterials in the photonic nanofluid provides higher full-spectrum absorption performance due to the photonic superposition of local surface plasmon resonance, surface plasmon resonance, and gap resonance at different wavelengths, as shown in Figure 7b [52,74]. The solar absorption efficiency of the dimer nanofluid improves by 21.2% compared to the blended nanofluid at the same number of spheres and rods. The various locations of spheres in the synthesis process of the dimer system result in a slight red shift or blue shift, which enhances the solar thermal conversion due to an increase in the broadband absorption spectrum.

Metal and transition metal nanoparticles show the localized surface plasmon resonance effect, and carbon nanomaterials have unique spectral absorption behaviors. The combined application of various nanomaterials offers excellent photothermal performance in the DASCs. In order to prepare ethylene glycol-based nanofluids with a significant broadband absorption in the visible and near-infrared spectrum range, plasmonic bimetallic Ag-Au alloy nanoparticles are loaded on ZIF-8-derived nitrogen-doped graphitic polyhedrons (ZNGs) at a lower concentration, corresponding to a photothermal conversion efficiency of 74.35% [75]. For the MWCNT-SiO_2_/Ag binary nanofluid, the solar thermal conversion and thermal conduction are improved due to the high absorption of MWCNTs in the infrared spectra and strong absorption peaks of SiO_2_/Ag nanoparticles within visible spectra [76]. When the reduced graphene oxides are decorated with silver nanoparticles, hybrid nanofluids show superior solar absorptance due to the plasmonic effect of the silver nanoparticles and the high thermal conductivity of graphene nanosheets [77,78]. It has been confirmed that the optical absorption property of titanium nitride (TiN) is superior to that of traditional materials, such as carbon nanotubes, graphene, gold (Au), silver (Ag), and metal sulfide (CuS) [69]. The nanoparticle transition metal nitrides, such as TiN, ZrN, and HfN nanospheres, exhibit small scattering cross-sections and broad absorption peaks, which match the solar radiation spectrum well [79]. Based on the synergy between the localized surface plasmon resonance effect and high thermal conductivity, the maximum photothermal conversion efficiency of the plasmonic nanofluids is 66.74%, which is 23% higher than that of pure water [80].

The thermal efficiency of the direct-absorption parabolic-trough solar collector (DAPTSC) with nanofluids is 5–10% higher than the conventional surface-based parabolic-trough solar collector (SBPTSC) [81]. However, the cost is high for most nanomaterials with a surface plasmon resonance effect. Although various nanoparticle structures have been theoretically designed to improve the solar absorption properties of plasma nanofluids, it is difficult to synthesize these complex structures. Efforts are needed to control nanoparticle parameters experimentally accurately, e.g., size and shape. To address these issues, researchers are exploring cost-effective synthesis methods, such as solution-based and green synthesis techniques, as well as the use of alternative materials, including non-precious metals and alloys. Additionally, advancements in automated production and scaling up manufacturing processes are expected to reduce costs. By optimizing synthesis techniques and expanding the range of applications for plasmonic nanomaterials, these efforts aim to make them more commercially viable and widely applicable.

### 2.4. Nanophase Change Slurry

**Figure 8:**
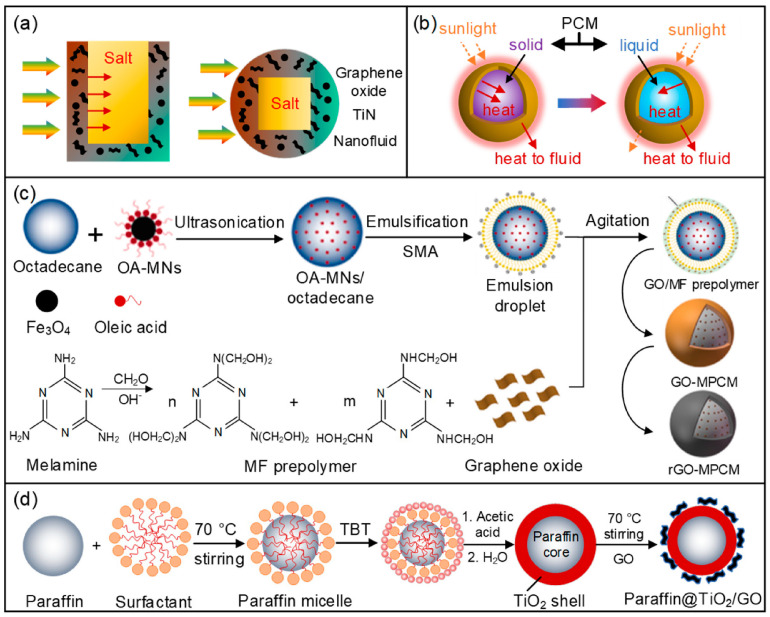
Nano-phase change material in DASCs: (**a**) ternary mixed molten salt [82], (**b**) ordinary phase change microcapsule [84], (**c**) magnetic nanoparticle-modified phase change microcapsule [85], and (**d**) GO-modified phase change microcapsule [86].

Thermal storage is a vital solution to the problems of the intermittence and instability of solar energy supply. For continuous heat output, an energy retention rate of 51.7% and a solar thermal efficiency of 56.5% are achieved using the GO-TiN/oil nanofluid as the working fluid and the ternary mixed molten salt as the heat storage core, as shown in Figure 8a [82]. Due to the large heat capacity of the energy storage medium and the excellent fluidity of the heat transfer fluid, the phase change slurry presents a promising solution for thermal applications in direct absorption solar collectors [83]. The direct absorption solar collector with a phase change slurry shows a higher photothermal conversion efficiency than other solar collectors, as shown in Figure 8b [84].

The nanofluid with microencapsulated phase change materials (MPCMs) is further considered to achieve excellent optical absorption properties with improved temperature stability. The temperature rise in the MPCM-MWCNT hybrid slurry is lower than that of the MWCNT nanofluid due to the high reflectivity of the MPCM surface and PCM melting-induced heat absorption [87]. Figure 8c shows the preparation of magnetic MPCM modified by reduced graphene oxide [85]. Magnetic nanoparticle-modified PCMs in a slurry present excellent controllability and recyclability by employing external magnetic fields [88,89]. This is because an external magnetic field enables precise manipulation and localization of the PCM slurry, thereby allowing on-demand transport and controlled heat storage/release. In addition, the soft-magnetic or superparamagnetic nature of the nanoparticles ensures rapid magnetization and demagnetization with negligible remanence, which facilitates efficient magnetic separation, recovery, and re-dispersion of the slurry, thus maintaining recyclability over repeated thermal cycles. Traditional microencapsulated PCM melamine resin is unfavorable for optical capture and heat conduction due to its low thermal conductivity and poor optical absorption. It is a potential solution for thermal applications of MPCMs in direct absorption solar collectors by coating nano-layer materials on the MPCMs or adopting nanomaterials as shells of PCMs. A novel microencapsulated phase change material is synthesized by encapsulating paraffin with a GO-modified TiO_2_ shell based on the in situ hydrolysis and polycondensation of tetrabutyl titanate, as shown in Figure 8d [86].

### 2.5. Comparative Analysis

In direct absorption solar collectors, the performance of nanofluids is critical for improving thermal efficiency and energy conversion. To evaluate the practical applicability of different nanofluid types, it is essential to assess key performance indicators, including efficiency, stability, cost, toxicity, and concentration range. These parameters significantly influence the thermal conductivity, operational stability, and economic feasibility of nanofluids within the collector system. The following Table 3 provides a comparative analysis of the performance of various nanofluid types in these aspects, highlighting their strengths and limitations in the context of direct absorption solar collectors, and offering valuable insights for their selection in practical applications.

## 3. Opportunities and Challenges of Nanofluid Applications

In the field of photothermal conversion, nanomaterials are dispersed in a base fluid through physical or chemical methods to create nanofluids. These nanofluids, acting as materials that absorb solar radiation, enhance both thermal conductivity and light absorption properties. However, several key challenges must be addressed in future research to fully realize the potential of nanofluids in photothermal applications.

### 3.1. Challenges

The application of nanofluids in photothermal conversion faces several critical challenges, particularly related to their environmental impact, economic feasibility, and safety concerns. These issues must be addressed to ensure their sustainable use in practical applications.

#### 3.1.1. Environmental Hazards

One significant environmental concern is the potential release of nanoparticles during the production and disposal phases of nanofluid life cycles. Nanoparticles possess unique physicochemical properties that, when released into the environment, can alter environmental media such as air, water, and soil. The accumulation of nanoparticles in ecosystems may adversely affect microbial communities, aquatic life, and soil organisms due to their high reactivity and toxicity. Furthermore, during the operational phase, the risk of leakage or dispersion of nanoparticles into the environment cannot be fully avoided. Nanoparticles in cooling or lubricating fluids can enter natural systems, leading to their accumulation and secondary transformations within ecosystems, thereby altering their bioavailability and toxicity.

The disposal and recycling of nanofluids remain underdeveloped, with many waste products potentially being discharged without adequate treatment. This increases the risk of long-term pollution and the persistence of nanoparticles in the environment, particularly those made of carbon-based materials and certain metal oxide nanoparticles, which have significant environmental persistence and bioaccumulation potential. These factors highlight the need for more comprehensive systems for nanofluid recycling, degradation, and disposal, alongside the establishment of environmental emission standards to mitigate these risks.

#### 3.1.2. Economic Barriers: Implementation Costs

The economic feasibility of nanofluids for widespread commercial use is another critical challenge. The high cost of obtaining and stabilizing nanoparticles significantly limits the scalability of nanofluid-based technologies. Nanoparticle production typically involves costly methods such as chemical vapor deposition, laser ablation, or high-energy milling, which are energy-intensive and expensive. Moreover, maintaining the stability of nanoparticles in the fluid phase—through the use of surfactants and stabilizers—further increases costs. These surfactants, while essential for preventing nanoparticle aggregation, add an additional financial burden, as their efficiency in stabilizing nanoparticles decreases under high temperatures and pressures, conditions often encountered in photothermal conversion systems.

The increased viscosity and pumping power requirements of nanofluids further complicate their large-scale use, making them less cost-effective compared to conventional fluids. For the commercialization of nanofluid-based technologies, it is crucial to develop more cost-effective methods for nanoparticle synthesis, stabilization, and the management of fluid properties.

#### 3.1.3. Long-Term Stability Under Operational Conditions

A major bottleneck in the application of nanofluids is their long-term stability under the demanding conditions of photothermal conversion systems. While short-term or intermittent testing has demonstrated the photothermal conversion efficiency of nanofluids, these tests fail to replicate the prolonged exposure to high temperatures, pressures, and UV radiation that occur in real-world applications. Under such extreme conditions, nanoparticles tend to aggregate, leading to sedimentation and a reduction in the nanofluid’s thermal and optical properties.

As the nanofluid operates over extended periods, its reusability diminishes, and aggregated nanoparticles cause increased wear and tear on equipment. This phenomenon is particularly problematic compared to single-species nanoparticles, which may perform more reliably over time. Moreover, the understanding of how nanoparticle aggregation affects the optical and thermal properties of nanofluids remains limited [90]. Future research is needed to elucidate the relationship between nanoparticle aggregation and the resultant changes in fluid properties to improve the long-term stability of nanofluids in real-world applications.

#### 3.1.4. Toxicity and Occupational Safety

Toxicological studies have shown that metal oxide and carbon-based nanoparticles can induce the generation of reactive oxygen species, leading to cellular damage, inflammation, and other adverse effects. Long-term exposure to nanofluids may also result in genetic toxicity and organ accumulation. Occupational health risks, particularly for workers involved in the production, handling, or disposal of nanofluids, have not been fully assessed, and more research is needed to clarify these risks.

The toxicity of nanofluids is highly dependent on the size, shape, concentration, and surface modifications of the nanoparticles. These variables complicate the establishment of uniform toxicity standards, making it difficult to assess the safety of nanofluids across different applications. Furthermore, the release of nanoparticles into the environment could harm microbial communities and aquatic organisms, disrupting ecosystems and increasing the risk of ecological imbalances.

The safety of nanofluids also faces challenges related to the lack of established guidelines for their handling, especially regarding long-term low-dose exposure, environmental releases during operations, and disposal or recycling of nanofluids. The implementation of safety protocols and the development of regulatory standards are necessary to mitigate occupational and environmental risks.

#### 3.1.5. Limitations of Current Nanofluid Models

Many numerical models for nanofluids rely on simplifying assumptions, such as treating nanofluids as homogeneous mixtures or neglecting the complex effects of nanoparticle aggregation. These models often fail to account for the dynamic behavior of nanoparticles under operational conditions, where aggregation and dispersion can significantly affect the thermal properties of the fluid. Additionally, factors such as temperature-dependent viscosity, particle-particle interactions, and the impact of surface modifications on thermal conductivity are often oversimplified or ignored.

Another limitation is the lack of accurate models that integrate both the macroscopic behavior of the nanofluid (such as heat transfer in the collector) and the microscopic interactions at the nanoparticle level. While models have been developed to simulate heat transfer in nanofluids, these often rely on generalized correlations that may not accurately predict the performance in specific solar collector designs or under varying operational conditions. The challenge is to incorporate these factors into more comprehensive models that can predict real-world behavior with greater precision.

Numerical simulations of nanofluid performance in solar collectors tend to focus on individual aspects, such as the effect of nanoparticle concentration or temperature, but often fail to offer a holistic view that includes the interaction of all relevant parameters (e.g., fluid dynamics, electromagnetic effects, and entropy generation) [91]. There is also a need for predictive tools that account for the long-term behavior of nanofluids, considering factors like particle agglomeration, thermal degradation, and the economic and environmental impacts over time.

### 3.2. Directions for Further Research

The following suggested research directions aim to address the current challenges in nanofluid technology and further enhance its application in photothermal conversion systems. These areas of focus are essential for advancing the performance, sustainability, and commercial viability of nanofluid-based technologies.

#### 3.2.1. Life Cycle Assessment (LCA) of Nanofluids in Solar Systems

Life cycle assessments (LCA) are critical for understanding the full environmental and economic impact of nanofluids in solar systems. Future research should focus on evaluating the environmental footprint of nanofluid production, usage, and disposal, including the impact of nanoparticle synthesis methods, transportation, and waste management. This assessment should also explore the economic costs of nanofluid-based solar systems compared to conventional systems, considering factors like the cost of nanoparticles, production processes, and system maintenance. Additionally, an LCA could help identify the most sustainable nanofluid formulations and usage strategies for real-world applications.

#### 3.2.2. Research on Hybrid Nanofluids in Real-World Conditions

Hybrid nanofluids, which combine different types of nanoparticles to leverage their complementary properties, represent an exciting avenue for improving the performance of solar thermal systems. Research on hybrid nanofluids should explore the synergistic effects of nanoparticles with optical, magnetic, and thermal storage properties, investigating their behavior under real-world operating conditions. This research should focus on improving the stability and heat transfer efficiency of these hybrid systems in actual solar collectors, addressing challenges such as nanoparticle dispersion, long-term stability, and scalability.

#### 3.2.3. Development of Predictive Models for Nanofluids in DASCs

The development of predictive models that combine the optical, thermal, and stability properties of nanofluids is crucial for optimizing their performance in direct absorption solar collectors (DASCs). Future models should integrate the interplay between nanoparticle concentration, shape, size, and surface modifications with thermal conductivity, heat transfer rates, and the overall stability of nanofluids. These models could significantly improve the efficiency of solar systems by providing better predictions of nanofluid behavior under varying operational conditions, ultimately leading to more effective design and application strategies.

#### 3.2.4. Optimization of Protective Coatings and Surfactants

To enhance the durability and reduce the toxicity of nanofluids, further research should be dedicated to optimizing protective coatings and surfactants. Protective coatings can prevent nanoparticle aggregation and degradation over time, improving the long-term stability of nanofluids in solar thermal systems. In parallel, surfactants need to be optimized to ensure they remain effective under high temperatures and pressures without introducing additional toxicity or environmental risks. Research should also explore the use of eco-friendly surfactants and coatings to reduce the environmental impact of nanofluid-based systems.

#### 3.2.5. Integration of DASCs with Other Renewable Energy Technologies

Integrating direct absorption solar collectors (DASCs) with other renewable energy technologies, such as photovoltaic-thermal (PVT) systems, could create hybrid systems with enhanced overall efficiency. Research in this area should investigate how nanofluids can be optimized for dual-function systems that simultaneously generate electricity and heat. This integration could lead to more efficient energy production, combining the benefits of both photovoltaic and solar thermal systems, and offering solutions to intermittency issues associated with renewable energy sources.

#### 3.2.6. Modeling and Simulation of Nanofluid Behavior

In addition to the experimental research, developing advanced simulation models is essential for predicting the performance of nanofluid-based systems in diverse real-world conditions. These models would simulate the behavior of nanofluids under varying environmental factors (e.g., temperature, radiation, flow conditions) and predict their long-term performance. This predictive capability will be crucial for designing more efficient solar collectors and understanding the underlying mechanisms that drive nanofluid behavior, ultimately aiding in the scaling and commercialization of nanofluid-based solar systems.

## 4. Conclusions

Compared to surface absorption solar collectors, direct absorption solar collectors are more effective in reducing energy loss. Surface absorption solar collectors rely on surface coating to absorb solar radiation, whereas direct absorption solar collectors utilize a heat transfer medium to absorb solar radiation. Numerous studies have demonstrated that incorporating nanofluids, which exhibit enhanced thermal properties such as thermal conductivity, significantly improve the efficiency of direct solar absorption collectors [92]. Researchers have identified the thermal conductivity of the working medium as the primary factor determining the efficiency of the direct absorption collector, with the thermal performance of the working medium serving as a secondary factor. Key conclusions from the review of nanofluid applications in direct absorption solar collectors include:

• Among common working fluids, the mixture of EG and water is considered the most suitable base fluid for anti-icing at night. While thermal oil shows high thermal conductivity, it is susceptible to thermal oxidation degradation under solar radiation. Conversely, water is the most widely used base fluid, and many reports have demonstrated the high photothermal conversion efficiency of water-based nanofluids.
• The nanofluid concentration strongly affects the thermal efficiency of the collector. Increasing the nanoparticle concentration results in a higher extinction coefficient for the nanofluid, a higher collector outlet temperature, improved energy efficiency, and enhanced exergy efficiency of the system. However, the increase in nanoparticle concentration also results in larger heat loss to the ambient. A critical particle concentration range exists that achieves the optimal photothermal conversion performance.
• The morphology of nanoparticles affects the extinction coefficient of nanofluids, with sharp-edged morphologies exhibiting excellent optical absorption characteristics.
• Nanoparticle size has a slight effect on the extinction coefficient within a specified range of spectral bands.
• In direct absorption solar collectors, the photothermal conversion efficiency can be ranked from highest to lowest in the order of metal nanofluids, carbon-based nanofluids, metal oxide nanofluids, and non-metal oxide nanofluids.
• Multivariate nanofluids offer advantages in absorbing solar spectra. A single nanoparticle usually exhibits only one spectral absorption peak. The specific performance improvement depends on various factors, such as type, concentration, ratio, and morphology of nanoparticles.
• Composite nanoparticles synthesized with multiple elements show high full-spectrum absorption performance. The optical absorption properties of nanofluids are not solely based on the direct addition of single particles, regardless of whether nanofluids are directly mixed or prepared by composite particles. The photonic superposition of local surface plasmon resonance, surface plasmon resonance propagation, and gap resonances at different wavelengths contributes to the difference in optical absorption performance.
• Al_2_O_3_ and CuO are commonly used metal oxide nanoparticles for preparing nanofluids as heat-absorbing media in direct absorption solar collectors. The average thermal efficiency improvement typically falls in the range of 10% to 40%. CuO nanoparticles show a higher extinction coefficient compared to Al_2_O_3_ nanoparticles.
• The thermomagnetic convection of magnetic nanofluids improved the utilization of non-sustainable solar energy. The photothermal conversion performance of magnetic nanofluids can be further enhanced through magnetic field induction, resulting in a 13% to 75% increase in convective heat transfer performance.
• Carbon nanomaterials, such as carbon nanotubes and graphene, exhibit excellent optical absorption and thermal conduction properties, resulting in a more than 170% increase in photothermal conversion efficiency. Comparatively, when nanofluids are prepared from common carbon nanomaterials such as carbon black and soot, the improvement in photothermal conversion efficiency of direct absorption solar collectors is below 100%.
• Surface chemical treatment can alter the hydrophilic/oleophilic characteristics of carbon nanomaterials, thereby improving their dispersion performance. However, surface treatment of carbon nanomaterials may potentially reduce thermal conductivity.
• The phase change slurry effectively maintains temperature uniformity within the collector. However, the absorption characteristics and heat conduction properties decrease due to the high reflectance and low thermal conductivity of shell materials. Combining phase change microcapsules with nanofluids enables energy storage and absorption of solar radiation.
• The application of plasmonic nanofluids in direct absorption solar collectors enhances optical absorption through the surface plasmon resonance effect, accelerates heat transfer due to their high thermal conductivity, and reduces operating costs by maintaining low particle concentrations.
• Core–shell plasmonic nanofluids achieve long-term stability over three years. The performance of the core–shell structure is affected by the shell structure and the number of layers.

## Figures and Tables

**Table 1:** Summary of studies for thermal performance of hybrid carbon/nitrogen nanomaterial-based nanofluid in DASCs.

Authors (Year)	Nanoparticle/Base Fluid	Particle Size	Remarks
Wang et al. (2019) [44]	α-Fe_2_O_3_-graphene oxide/EG	α-Fe_2_O_3_ nanorod: length of 60–500 nm; diameter of 30–80 nm	A 56.8% photothermal conversion efficiency for α-Fe_2_O_3_-graphene oxide/EG nanofluids with magnetic fields, 14.5% higher than non-external rotating magnetic field nanofluids with a 0.007 wt% RGO
Sreehari et al. (2020) [34]	Carbon soot/water	50 nm	A 50% thermal efficiency for carbon soot nanofluid; 70% of the incident energy absorption for carbon soot nanofluid
Li et al. (2020) [45]	SiC-MWCNTs/EG	MWCNTs: length of ~30 um; SiC: 40 nm	99.9% solar energy absorption for 0.5 wt% SiC-MWCNTs nanofluids at 1 cm path length; 97.3% solar-thermal conversion efficiency for 1 wt% SiC-MWCNTs nanofluid at 10 min, 48.6% higher than that of pure EG
Huang et al. (2020) [46]	Hydroxylated graphene/EG-water	Number of layers of less than 10; Thickness of 0.55–3.74 μm; diameter of 0.5–3 μm	A 73.5% instantaneous heat collection efficiency for 0.007 wt% graphene nanofluid at 30 °C, 42.22% higher than the base liquid
Mashhadian et al. (2021) [42]	Al_2_O_3_, MWCNTs/water	MWCNTs: length 5–10 μm; diameter 20–30 nm; Al_2_O_3_: 40 nm	A 197.1% increase in thermal efficiency of the solar collector by using hybrid nanofluids compared to water
Struchalin et al. (2021) [8]	MWCNTs/water-ethanol	Inner diameter 13.3 ± 0.45 nm; external diameter 49.3 ± 0.45 nm or 72.0 ± 0.45 nm; maximum length 5 μm	An increase of 5.8–37.9% in the efficiency of the solar collector for 0.01 wt% MWCNTs nanofluid relative to an equivalent geometry with surface absorption
Hazra et al. (2021) [38]	Boron nitride (BN)-carbon black (CN)/ethylene glycol (EG)	70 nm	An absorbed energy fraction of 98.92% for 90 ppm/15 ppm BN/CB nanofluid; An enhancement of 34.55% photo-thermal conversion efficiency for 90 ppm/15 ppm BN/CB nanofluid after a 1200 s exposure to 437 W/m^2^ incident irradiance

**Table 3:** Comparative analysis of the performance of different types of nanofluids in DASCs.

Nanofluid Type	Enhanced Thermal Efficiency	Stability	Costs	Toxicity	Suggested Concentration
Oxide nanomaterial-based nanofluid	13–75%	Low to moderate	Moderate	Low	0.5–2.0 wt.%
Carbon/Nitrogen nanomaterial-based nanofluid	14–189%	High	Low	Low	0.01–1.0 wt.%
Plasmonic nanofluid	20–240%	High	High	Moderate	0.01–1.0 wt.%
Nanophase change slurry	20–40%	High	Moderate	Low	10–20 wt.%

## Data Availability

Data are available in a publicly accessible repository.

## References

[1] Choi S.U.S., Singer D.A., Wang H.P. (1995). Developments and Applications of Non-Newtonian Flows. ASME Fed..

[2] Sun L., Yang L., Zhao N., Song J., Li X., Wu X. (2022). A Review of Multifunctional Applications of Nanofluids in Solar Energy. Powder Technol..

[3] Nobrega G., de Souza R.R., Gonçalves I.M., Moita A.S., Ribeiro J.E., Lima R.A. (2022). Recent Developments on the Thermal Properties, Stability and Applications of Nanofluids in Machining, Solar Energy and Biomedicine. Appl. Sci..

[4] Lim A.E., Goh S. (2023). Effect of Microchannel Diameter on Electroosmotic Flow Hysteresis. Energies.

[5] Moravej M., Bozorg M.V., Guan Y., Li L.K.B., Doranehgard M.H., Hong K., Xiong Q. (2020). Enhancing the Efficiency of a Symmetric Flat-Plate Solar Collector via the Use of Rutile TiO_2_-Water Nanofluids. Sustain. Energy Technol. Assess..

[6] Sharafeldin M.A., Gróf G. (2018). Evacuated Tube Solar Collector Performance Using CeO_2_/Water Nanofluid. J. Clean. Prod..

[7] Bellos E., Tzivanidis C. (2018). Thermal Analysis of Parabolic Trough Collector Operating with Mono and Hybrid Nanofluids. Sustain. Energy Technol. Assess..

[8] Struchalin P.G., Yunin V.S., Kutsenko K.V., Nikolaev O.V., Vologzhannikova A.A., Shevelyova M.P., Gorbacheva O.S., Balakin B. (2021). V Performance of a Tubular Direct Absorption Solar Collector with a Carbon-Based Nanofluid. Int. J. Heat Mass Transf..

[9] Struchalin P.G., Kuzmenkov D.M., Yunin V.S., Wang X., He Y., Balakin B. (2022). V Hybrid Nanofluid in a Direct Absorption Solar Collector: Magnetite vs. Carbon Nanotubes Compete for Thermal Performance. Energies.

[10] Muraleedharan M., Singh H., Suresh S., Udayakumar M. (2016). Directly Absorbing Therminol-Al_2_O_3_ Nano Heat Transfer Fluid for Linear Solar Concentrating Collectors. Sol. Energy.

[11] Behura A.K., Gupta H.K. (2020). Efficient Direct Absorption Solar Collector Using Nanomaterial Suspended Heat Transfer Fluid. Mater. Today-Proc..

[12] Valizade M., Heyhat M.M., Maerefat M. (2019). Experimental Comparison of Optical Properties of Nanofluid and Metal Foam for Using in Direct Absorption Solar Collectors. Sol. Energy Mater. Sol. Cells.

[13] Joseph A., Thomas S. (2021). A Hybrid Approach of Surface and Direct Absorption of Solar Radiation on Parabolic Solar Collector. J. Clean. Prod..

[14] Khalil A., Amjad M., Noor F., Hussain A., Nawaz S., Bandarra Filho E.P., Du X. (2020). Performance Analysis of Direct Absorption-Based Parabolic Trough Solar Collector Using Hybrid Nanofluids. J. Braz. Soc. Mech. Sci. Eng..

[15] Pandi K., Venkatachalapathy S., Suresh S. (2022). Experimental Investigation on Low Concentration Nanofluids with Fresnel Lens and Evacuated Tubes for Solar Applications. Int. J. Appl. Ceram. Technol..

[16] Bonab H.B., Javani N. (2019). Investigation and Optimization of Solar Volumetric Absorption Systems Using Nanoparticles. Sol. Energy Mater. Sol. Cells.

[17] Ham J., Shin Y., Cho H. (2021). Thermal Performance of Solar Collector Based on Volumetric Absorption Harvesting Method Using Fe_3_O_4_ Nanofluid. High Temp.-High Press..

[18] Narankhishig Z., Ham J., Lee H., Cho H. (2021). Convective Heat Transfer Characteristics of Nanofluids Including the Magnetic Effect on Heat Transfer Enhancement—A Review. Appl. Therm. Eng..

[19] Sani E., Martina M.R., Salez T.J., Nakamae S., Dubois E., Peyre V. (2021). Multifunctional Magnetic Nanocolloids for Hybrid Solar-Thermoelectric Energy Harvesting. Nanomaterials.

[20] Balakin B.V., Zhdaneev O.V., Kosinska A., Kutsenko K. (2019). V Direct Absorption Solar Collector with Magnetic Nanofluid: CFD Model and Parametric Analysis. Renew. Energy.

[21] Wang D., Liang W., Zheng Z., Jia P., Yan Y., Xie H., Wang L., Yu W. (2021). Highly Efficient Energy Harvest via External Rotating Magnetic Field for Oil Based Nanofluid Direct Absorption Solar Collector. Green Energy Environ..

[22] Hosseini S.M.S., Ayar D., Talebizadeh A., Kamyabi M. (2022). An Experimental Investigation on the Solar-Thermal Energy Conversion Performance of Fe2O3 Nanofluid with the Focus on Nanoparticles Shape and Concentration. Int. J. Energy Res..

[23] Karami M., Delfani S., Esmaeili M. (2019). Effect of V-Shaped Rib Roughness on the Performance of Wnanofluid-Based Direct Absorption Solar Collectors. J. Therm. Anal. Calorim..

[24] Sattar A., Farooq M., Amjad M., Saeed M.A., Nawaz S., Mujtaba M.A., Anwar S., El-Sherbeeny A.M., Soudagar M.E.M., Bandarra Filho E.P. (2020). Performance Evaluation of a Direct Absorption Collector for Solar Thermal Energy Conversion. Energies.

[25] Luo Z., Wang C., Wei W., Xiao G., Ni M. (2014). Performance Improvement of a Nanofluid Solar Collector Based on Direct Absorption Collection (DAC) Concepts. Int. J. Heat Mass Transf..

[26] Ham J., Shin Y., Cho H. (2019). Optical-Thermal Properties of an MWCNT/EG Nanofluid Intended as the Working Fluid in a Direct Absorption Solar Collector. High Temp.-High Press..

[27] Mahbubul I.M., Khan M.M.A., Ibrahim N.I., Ali H.M., Al-Sulaiman F.A., Saidur R. (2018). Carbon Nanotube Nanofluid in Enhancing the Efficiency of Evacuated Tube Solar Collector. Renew. Energy.

[28] Jeong M.G., Kim J.B., Qin C., Lee J., Lee B.J. (2021). Synthesis of Therminol-Graphite Nanofluids and Photo-Thermal Conversion Properties. Int. J. Energy Res..

[29] Sa-ard W.C., Fawcett D., Fung C.C., Chapman P., Rattan S., Poinern G.E.J. (2021). Synthesis, Characterisation and Thermo-Physical Properties of Highly Stable Graphene Oxide-Based Aqueous Nanofluids for Potential Low-Temperature Direct Absorption Solar Applications. Sci. Rep..

[30] Li Z., Kan A., Wang K., He Y., Zheng N., Yu W. (2022). Optical Properties and Photothermal Conversion Performances of Graphene Based Nanofluids. Appl. Therm. Eng..

[31] Xu X., Xu C., Liu J., Fang X., Zhang Z. (2019). A Direct Absorption Solar Collector Based on a Water-Ethylene Glycol Based Nanofluid with Anti-Freeze Property and Excellent Dispersion Stability. Renew. Energy.

[32] Guo J., Wang F., Li S., Wang Y., Hu X., Zu D., Shen Y., Li C. (2022). Enhanced Optical Properties and Light-to-Heat Conversion Performance of Ti_3_C_2_/[BMIM]BF4 Nanofluids Based Direct Absorption Solar Collector. Sol. Energy Mater. Sol. Cells.

[33] Li X., Chang H., Zeng L., Huang X., Li Y., Li R., Xi Z. (2020). Numerical Analysis of Photothermal Conversion Performance of MXene Nanofluid in Direct Absorption Solar Collectors. Energy Convers. Manag..

[34] Sreehari S., Joseph A., Thomas S. (2020). Development of a Low Cost Nanofluid Based Direct Absorption Solar Collector. Mater. Today-Proc..

[35] Joseph A., Thomas S. (2022). Energy, Exergy and Corrosion Analysis of Direct Absorption Solar Collector Employed with Ultra-High Stable Carbon Quantum Dot Nanofluid. Renew. Energy.

[36] Chen X., Xiong Z., Chen M., Zhou P. (2022). Ultra-Stable Carbon Quantum Dot Nanofluids for Direct Absorption Solar Collectors. Sol. Energy Mater. Sol. Cells.

[37] Kumar P.G., Vigneswaran S., Meikandan M., Sakthivadivel D., Salman M., Thakur A.K., Sathyamurthy R., Kim S.C. (2022). Exploring the Photo-Thermal Conversion Behavior and Extinction Coefficient of Activated Carbon Nanofluids for Direct Absorption Solar Collector Applications. Environ. Sci. Pollut. Res..

[38] Hazra S.K., Michael M., Nandi T.K. (2021). Investigations on Optical and Photo-Thermal Conversion Characteristics of BN-EG and BN/CB-EG Hybrid Nanofluids for Applications in Direct Absorption Solar Collectors. Sol. Energy Mater. Sol. Cells.

[39] Kim H., Ham J., Cho H. (2022). Evaluation on the Photothermal Conversion Performance of SiC Nanofluid for a Direct Absorption Solar Collector. Int. J. Nanotechnol..

[40] Inayat U., Iqbal S., Manzoor T., Zia M.F. (2021). Numerical Investigation of Heat Transfer on Unsteady Hiemenz Cu-Water and Ag-Water Nanofluid Flow over a Porous Wedge Due to Solar Radiation. Appl. Sci..

[41] Yu X., He L., Liu R., Guan H., Ge C., Zhang X. (2021). Preparation and Photothermal Conversion of H-BN/CuO Nanofluids. ChemistrySelect.

[42] Mashhadian A., Heyhat M.M., Mahian O. (2021). Improving Environmental Performance of a Direct Absorption Parabolic Trough Collector by Using Hybrid Nanofluids. Energy Convers. Manag..

[43] Jin X., Lin G., Jin H., Fu Z., Sun H. (2021). Experimental Research on the Selective Absorption of Solar Energy by Hybrid Nanofluids. Energies.

[44] Wang D., Jia Y., He Y., Wang L., Xie H., Yu W. (2019). Photothermal Efficiency Enhancement of a Nanofluid-Based Direct Absorption Solar Collector Utilizing Magnetic Nano-Rotor. Energy Convers. Manag..

[45] Li X., Zeng G., Lei X. (2020). The Stability, Optical Properties and Solar-Thermal Conversion Performance of SiC-MWCNTs Hybrid Nanofluids for the Direct Absorption Solar Collector (DASC) Application. Sol. Energy Mater. Sol. Cells.

[46] Huang J., Chen Z., Du Z., Xu X., Zhang Z., Fang X. (2020). A Highly Stable Hydroxylated Graphene/Ethylene Glycol-Water Nanofluid with Excellent Extinction Property at a Low Loading for Direct Absorption Solar Collectors. Thermochim. Acta.

[47] Kumar S., Sharma V., Samantaray M.R., Chander N. (2020). Experimental Investigation of a Direct Absorption Solar Collector Using Ultra Stable Gold Plasmonic Nanofluid under Real Outdoor Conditions. Renew. Energy.

[48] Mallah A.R., Zubir M.N.M., Alawi O.A., Kazi M.S.N., Ahmed S.M., Oon C.S., Mohamad A.B. (2020). An Innovative, High-Efficiency Silver/Silica Nanocomposites for Direct Absorption Concentrating Solar Thermal Power. Int. J. Energy Res..

[49] Duan H., Tang L., Zheng Y., Xu C. (2018). Effect of Plasmonic Nanoshell-Based Nanofluid on Efficiency of Direct Solar Thermal Collector. Appl. Therm. Eng..

[50] Qin C., Kang K., Lee I., Lee B.J. (2018). Optimization of the Spectral Absorption Coefficient of a Plasmonic Nanofluid for a Direct Absorption Solar Collector. Sol. Energy.

[51] Sreekumar S., Joseph A., Kumar C.S.S., Thomas S. (2020). Investigation on Influence of Antimony Tin Oxide/Silver Nanofluid on Direct Absorption Parabolic Solar Collector. J. Clean. Prod..

[52] Chen Z., Chen M., Yan H., Zhou P., Chen X. (2020). Enhanced Solar Thermal Conversion Performance of Plasmonic Gold Dimer Nanofluids. Appl. Therm. Eng..

[53] Mallah A.R., Kazi S.N., Zubir M.N.M., Badarudin A. (2018). Blended Morphologies of Plasmonic Nanofluids for Direct Absorption Applications. Appl. Energy.

[54] Yu X., Xuan Y. (2018). Investigation on Thermo-Optical Properties of CuO/Ag Plasmonic Nanofluids. Sol. Energy.

[55] Qin C., Kim J.B., Gonome H., Lee B.J. (2020). Absorption Characteristics of Nanoparticles with Sharp Edges for a Direct-Absorption Solar Collector. Renew. Energy.

[56] Walshe J., Amarandei G., Ahmed H., McCormack S., Doran J. (2019). Development of Poly-Vinyl Alcohol Stabilized Silver Nanofluids for Solar Thermal Applications. Sol. Energy Mater. Sol. Cells.

[57] Rativa D., Gómez-Malagón L.A. (2018). Colloidal Plasmonic Structures for Harvesting Solar Radiation. Renew. Energy.

[58] Wu Y., Zhou L., Du X., Yang Y. (2015). Optical and Thermal Radiative Properties of Plasmonic Nanofluids Containing Core-Shell Composite Nanoparticles for Efficient Photothermal Conversion. Int. J. Heat Mass Transf..

[59] Duan H., Chen R., Zheng Y., Xu C. (2018). Photothermal Properties of Plasmonic Nanoshell-Blended Nanofluid for Direct Solar Thermal Absorption. Opt. Express.

[60] Chen X., Wu D., Zhou P., Chen M., Yan H. (2021). Modeling the Solar Absorption Performance of Copper@Carbon Core-Shell Nanoparticles. J. Mater. Sci..

[61] Lee R., Kim J.B., Qin C., Lee H., Lee B.J., Jung G.Y. (2020). Synthesis of Therminol-Based Plasmonic Nanofluids with Core/Shell Nanoparticles and Characterization of Their Absorption/Scattering Coefficients. Sol. Energy Mater. Sol. Cells.

[62] Sharaf O.Z., Rizk N., Joshi C.P., Jaoude M.A., Al-Khateeb A.N., Kyritsis D.C., Abu-Nada E., Martin M.N. (2019). Ultrastable Plasmonic Nanofluids in Optimized Direct Absorption Solar Collectors. Energy Convers. Manag..

[63] Sharaf O.Z., Rizk N., Munro C.J., Joshi C.P., Waheed W., Abu-Nada E., Alazzam A., Martin M.N. (2021). Thermal Stability and Plasmonic Photothermal Conversion of Surface-Modified Solar Nanofluids: Comparing Prolonged and Cyclic Thermal Treatments. Energy Convers. Manag..

[64] Sharaf O.Z., Rizk N., Munro C.J., Joshi C.P., Anjum D.H., Abu-Nada E., Martin M.N., Alazzam A. (2021). Radiation Stability and Photothermal Performance of Surface-Functionalized Plasmonic Nanofluids for Direct-Absorption Solar Applications. Sol. Energy Mater. Sol. Cells.

[65] Shang L., Qu J., Wang Z., Zhang M., Li C. (2021). Optical Absorption Property and Photo-Thermal Conversion Performance of Ag@Al_2_O_3_ Plasmonic Nanofluids with Al2O3 Nano-Shell Fabricated by Atomic Layer Deposition. J. Mol. Liq..

[66] Joseph A., Sreekumar S., Kumar C.S.S., Thomas S. (2019). Optimisation of Thermo-Optical Properties of SiO_2_/Ag–CuO Nanofluid for Direct Absorption Solar Collectors. J. Mol. Liq..

[67] Wen J., Li X., Chen W., Liu J. (2021). Systematical Investigation on the Solar-Thermal Conversion Performance of TiN Plasmonic Nanofluids for the Direct Absorption Solar Collectors. Colloids Surf. A Physicochem. Eng. Asp..

[68] Mallah A.R., Zubir M.N.M., Alawi O.A., Kazi S.N., Ahmed W., Sadri R., Kasaeian A. (2022). Experimental Study on the Effects of Multi-Resonance Plasmonic Nanoparticles for Improving the Solar Collector Efficiency. Renew. Energy.

[69] Wang L., Zhu G., Wang M., Yu W., Zeng J., Yu X., Xie H., Li Q. (2019). Dual Plasmonic Au/TiN Nanofluids for Efficient Solar Photothermal Conversion. Sol. Energy.

[70] Farooq S., Vital C.V.P., Gomez-Malagon L.A., de Araujo R.E., Rativa D. (2020). Thermo-Optical Performance of Iron-Doped Gold Nanoshells-Based Nanofluid on Direct Absorption Solar Collectors. Sol. Energy.

[71] Jin X., Lin G., Zeiny A., Jin H., Bai L., Wen D. (2019). Solar Photothermal Conversion Characteristics of Hybrid Nanofluids: An Experimental and Numerical Study. Renew. Energy.

[72] Wang X., He Y., Chen M., Hu Y. (2018). ZnO-Au Composite Hierarchical Particles Dispersed Oil-Based Nanofluids for Direct Absorption Solar Collectors. Sol. Energy Mater. Sol. Cells.

[73] Kimpton H., Stulz E., Zhang X. (2020). Silver Nanofluids Based Broadband Solar Absorber through Tuning Nanosilver Geometries. Sol. Energy.

[74] Zeng J., Xuan Y. (2022). Direct Solar-Thermal Conversion Features of Flowing Photonic Nanofluids. Renew. Energy.

[75] Zhu G., Wang L., Bing N., Xie H., Yu W. (2019). Enhancement of Photothermal Conversion Performance Using Nanofluids Based on Bimetallic Ag-Au Alloys in Nitrogen-Doped Graphitic Polyhedrons. Energy.

[76] Zeng J., Xuan Y. (2018). Enhanced Solar Thermal Conversion and Thermal Conduction of MWCNT-SiO_2_/Ag Binary Nanofluids. Appl. Energy.

[77] Hong Z., Pei J., Wang Y., Cao B., Mao M., Liu H., Jiang H., An Q., Liu X., Hu X. (2019). Characteristics of the Direct Absorption Solar Collectors Based on Reduced Graphene Oxide Nanofluids in Solar Steam Evaporation. Energy Convers. Manag..

[78] Mehrali M., Ghatkesar M.K., Pecnik R. (2018). Full-Spectrum Volumetric Solar Thermal Conversion via Graphene/Silver Hybrid Plasmonic Nanofluids. Appl. Energy.

[79] Vital C.V.P., Farooq S., de Araujo R.E., Rativa D., Gomez-Malagon L.A. (2021). Numerical Assessment of Transition Metal Nitrides Nanofluids for Improved Performance of Direct Absorption Solar Collectors. Appl. Therm. Eng..

[80] Luo B., Han Y., Yang T., Li J., Wang H., Chen W., Li X. (2021). Systematic Investigating on the Broadband Solar Absorption and Photo-Thermal Conversion Performance of TiN@rGO Plasmonic Nanofluids. Colloids Surf. A Physicochem. Eng. Asp..

[81] Qin C., Kim J.B., Lee B.J. (2019). Performance Analysis of a Direct-Absorption Parabolic-Trough Solar Collector Using Plasmonic Nanofluids. Renew. Energy.

[82] Zhang H., Wang K., Yu W., Wang L., Xie H. (2021). Ternary Molten Salt Energy Storage Coupled with Graphene Oxide-TiN Nanofluids for Direct Absorption Solar Collector. Energy Build..

[83] Tian L., Liu J., Wu Z., Klemes J.J., Wang J. (2022). Experimental Study on Photovoltaic/Thermal System Performance Based on Microencapsulated Phase Change Material Slurry. Energy Sources Part A-Recovery Util. Environ. Eff..

[84] Ma F., Zhang P. (2019). Performance Investigation of the Direct Absorption Solar Collector Based on Phase Change Slurry. Appl. Therm. Eng..

[85] Gao G., Zhang T., Jiao S., Guo C. (2020). Preparation of Reduced Graphene Oxide Modified Magnetic Phase Change Microcapsules and Their Application in Direct Absorption Solar Collector. Sol. Energy Mater. Sol. Cells.

[86] Ji W., Cheng X., Chen S., Wang X., Li Y. (2021). Self-Assembly Fabrication of GO/TiO_2_@paraffin Microcapsules for Enhancement of Thermal Energy Storage. Powder Technol..

[87] Wang Z., Qu J., Zhang R., Han X., Wu J. (2019). Photo-Thermal Performance Evaluation on MWCNTs-Dispersed Microencapsulated PCM Slurries for Direct Absorption Solar Collectors. J. Energy Storage.

[88] Li F., Sun Z., Jiao S., Ma Y., Zhang Q., Zhou Y., Wen J., Liu Y. (2021). Preparation and Performance of Dual-Functional Magnetic Phase-Change Microcapsules. Chem. Asian J..

[89] Jiang F., Wang X., Wu D. (2016). Magnetic Microencapsulated Phase Change Materials with an Organo-Silica Shell: Design, Synthesis and Application for Electromagnetic Shielding and Thermal Regulating Polyimide Films. Energy.

[90] Jing D., Song D. (2017). Optical Properties of Nanofluids Considering Particle Size Distribution: Experimental and Theoretical Investigations. Renew. Sustain. Energy Rev..

[91] Huminic G., Huminic A. (2020). Entropy Generation of Nanofluid and Hybrid Nanofluid Flow in Thermal Systems: A Review. J. Mol. Liq..

[92] Javadi F.S., Saidur R., Kamalisarvestani M. (2013). Investigating Performance Improvement of Solar Collectors by Using Nanofluids. Renew. Sustain. Energy Rev..

